# The Role of Milk Components, Pro-, Pre-, and Synbiotic Foods in Calcium Absorption and Bone Health Maintenance

**DOI:** 10.3389/fnut.2020.578702

**Published:** 2020-09-23

**Authors:** Bolaji L. Ilesanmi-Oyelere, Marlena C. Kruger

**Affiliations:** ^1^School of Health Sciences, College of Health, Massey University, Palmerston North, New Zealand; ^2^Riddet Institute, Massey University, Palmerston North, New Zealand

**Keywords:** milk, probiotics, prebiotics, calcium absorption, bone health, gut microbiome

## Abstract

Increasing peak bone mass during adolescence and reducing bone loss in later life are two approaches to reduce the risk of osteoporosis with aging. Osteoporosis affects a large proportion of the elderly population worldwide and the incidence is increasing. Milk consumption is an accepted strategy in building peak bone mass and therefore may reduce the risk of osteoporosis. In childhood calcium, phosphorous, and growth factors are the important components to support bone growth but in adults the positive influence on bone density/maintenance may also be due to other bioactive proteins/peptides or lipids in milk acting directly in the gastrointestinal tract (GIT). Lactose has been known to increase calcium absorption; galactooligosaccharides (GOS) are derived from lactose and are non-digestible oligosaccharides. They have been shown to improve mineral balance and bone properties as well as causing increases in bifidobacteria in the gut, therefore a prebiotic effect. Supplementation with fortified milk and dairy products with added prebiotics, increased both calcium and magnesium absorption and caused some modulation of gut microbiota in animals and humans. Fermented milk is now also recognized to contain highly active components such as vitamins, peptides, oligosaccharides, and organic acids. In this review, the role of milk and milk components in improving calcium absorption and thereby supporting bone health is discussed. In addition, some reference is made to the significance of combining the inherent beneficial components from milk with fortificants/nutrients that will support bone health through adulthood. Novel data suggesting differences in diversity of the microbiota between healthy and osteoporotic women are provided.

## Introduction

Osteoporosis is a condition of skeletal fragility characterized by decreased bone mass and microarchitectural deterioration of the bone structure (1), with the consequence of an increased risk of fracture. Several factors can influence the development of osteoporosis with aging. Genetic background and ethnicity are non-modifiable determinants of bone health while nutrition and lifestyle are modifiable factors that can contribute to osteoporosis. Peak bone mass (PBM) is the greatest amount of bone an individual can attain and is reached in the late teens and early 20's. At the age of 18 usually 90% of PBM is reached and while nutrition and exercise have significant effects on the attainment of PBM, genetics has the strongest influence (2). While calcium is the most important nutrient for bone growth and reduction of bone resorption, the use of dairy products or milk may have further benefits due to bioactive components of milk and milk or dairy foods are also excellent vehicles for fortification. In this review the role of milk in increasing bone growth and reducing bone resorption is discussed followed by a review of further bioactive components of milk which may enhance the bone health benefits of milk/dairy.

## Milk and Bone Health in Children and Older Adults

Several randomized controlled trials in children and adults have used milk and milk products as the principal source of calcium, all showing positive effects (1). Early studies in Caucasian and Gambian children indicated that increasing milk intake may increase bone formation, and increase the levels of insulin growth factor—one (IGF-I), while supplementing with fortified milk with additional vitamin D resulted in significant increases in lumbar spine bone density compared to the controls (3). In contrast a meta-analysis as well as a review indicated that increased milk/ dairy intake may only be effective in children with a low habitual intake (2). A prospective study in children aged 5–10 years over 1 year however, reported a marginal significant effect on whole body (WB) bone mineral content and improvement in the WB *z*-score (2).

Iuliano and Hill (3) provided a critical appraisal of the evidence for a beneficial effect of dairy foods on bone density/ bone biomarkers throughout the lifespan. Several studies have shown significant effects of regular or fortified milk on bone biomarkers reducing bone resorption, but evidence of significant effects on bone density and reduction in fracture risk are less available. As discussed by Iuliano and Hill (3), to date no randomized controlled prospective studies have been executed on milk/ dairy foods investigating the anti-fracture effects. In general studies using either calcium supplementation or dairy foods had several flaws such as poor compliance, insufficient numbers of participants, shorter duration of the studies, and a significant drop-out rate which then reduced the opportunity to observe an antifracture effect. Observing a change in bone density or reduction in fracture risk may require a specific population to be studied and a longer trial period which may not be feasible or affordable.

## Components of Milk and Dairy Foods

The main components of dairy that have been shown to affect the human health are protein (from whey, casein, fragments), fat [saturated/unsaturated, short chain fatty acids (SCFA)], minerals (calcium, magnesium, phosphate), sodium, and sugars (lactose, galactose, glucose) as well as the components of the milk fat globule membrane (MFGM) (i.e., the biological membrane surrounding the lipid droplets in milk) (4). The final dairy product nutritional value tends to be determined by the milk-based source (age, mammalian-type, feed and lactation stage), processing/preparation type (storage condition, temperature and heat duration) as well as the fermentation and cultures used and any additives (5). Table 1 shows a list of controlled trials using dairy products to mitigate bone loss and support bone health in adults.

**Table 1:** Controlled trials on the effect of dairy products on bone turnover markers and bone mass^a^.

**Type of dairy**	**Number of subjects**	**Age^b^/Sex**	**Duration (months)**	**Outcome**	**References**
Fortified milk and yogurt, 3 servings per day	101	60.5 ± 0.7/F	12	Reduced PTH and CTX, increase in BMD	(6)
Milk	30	59.3 ± 0.3/F	1.5	Reduced PTH, CTX, PINP and osteocalcin	(7)
High-protein dairy	130	45.6 ± 8.9/F/M	12	Attenuated bone loss	(8)
Fortified fermented milk (175mL)	85	58.7 ± 0.3/F	0.5	Reduced excretion of nocturnal deoxypyridinoline	(9)
Skimmed soft cheese, 2 servings/d	37	84.8 ± 8.1/F	1	Reduced PTH, CTX and TRAP 5b, and increased 25(OH)D and IGF-1	(7)
Fortified milk	120	>55/F	4	Reduced PTH, CTX, PINP and osteocalcin	(10)
Milk (2 × 500 mL/d)	20	22.4 ± 2.4/F	3	Reduced PTH and CTX	(11)
Fortified milk	63	>55/F	3	Reduced CTX	(12)
Skimmed soft cheese, 2 servings (100 g/d)	71	56.6 ± 3.0/F	1.5	Reduced PTH, CTX and TRAP 5b, and increased IGF-1	(13)
Vitamin D and Calcium-fortified yogurt	89	85.5 ± 6.6/F	2	Reduced PTH, CTX and TRAP 5b	(14)

a *BMD, bone mineral density; PTH, parathyroid hormone; CTX, cross-linked telopeptide of type 1 collagen; PINP, procollagen type I N-propeptide; TRAP 5b, tartrate-resistant acid phosphatase; 25(OH)D, 25-hydroxyvitamin D*.

b *Mean ± SD*.

The health-promoting benefit and effects of probiotic, prebiotic and synbiotic food products have been researched mainly in animal models however, not much work has been done with humans. Research shows that probiotics, prebiotics and synbiotics can be beneficial for mineral absorption and bioavailability in bone health, modulation of growth factors, reduction in inflammatory cytokines and an antiarthritic effect, blood lipids and a protective effect on chronic inflammatory conditions such as gastritis, Crohn's disease, inflammatory bowel disease (IBD) and type II diabetes (15).

## The Impact of Milk Components (Milk Protein, Milk Fat and Milk Sugar) on Bone Health

### Milk Sugars

Milk is known as an important source of energy (from the milk sugars), high-quality protein and essential vitamins as well as minerals. Lactose malabsorption and/or intolerance have been linked to bone loss and fractures (16). D-galactose has been linked with inducing aging, oxidative stress and inflammation; however galactooligosaccharides and transgalactooligosaccharides are known to stimulate calcium absorption in humans (17) which is important for bone health. Lactose is a disaccharide carbohydrate which is a well-known nutrient that promotes intestinal calcium absorption in mammals. Hydrolyzed lactose forms simple sugars, glucose and galactose with organic acids which reduce the pH thereby enhancing the transport and absorption of calcium ions as calcium gluconate in the intestine (18). Meanwhile, if unhydrolyzed, lactose moves into the large intestine acting as a prebiotic where it stimulates the growth of health-promoting microbes such as *Bifidobacteria* (19). Lactose has also been reported to enhance the formation of non-digestible oligosaccharides (NDOs) such as transgalactooligosaccarides due to lactic acid bacteria growth.

### Milk Fats

Research into the effects of fatty acids on human health has identified that diets high in palmitic acids (C16:0), myristic (C14:0) and lauric (C12:0) are associated with increased risk of atherosclerosis (20), coronary heart diseases and obesity (21). However, the fat globules of milk contain short-chain and polyunsaturated fatty acids. Unsaturated fatty acids are regarded as “healthy fats” due to their impact on cholesterol levels in the blood (22). Polyunsaturated fatty acids (PUFAs) are known to have a more beneficial effect on cholesterol levels compared to MUFAs (23).

Reports has suggested that n−3 PUFA prevents cardiovascular disease and improve the immune response. Likewise, the anticancer and anti-atherogenic properties of oleic acid (C18:1) and linolenic acid (C18:3) have been reported (20). Research into the beneficial effects of n-6 PUFA has shown it ability to improve sensitivity to insulin thereby reducing the incidence of type 2 diabetes (24). Furthermore, the health-positive properties of conjugated linoleic acid cis9trans11 C18:2 (CLA) have been reported which include anticarcinogenic (25), anti-atherosclerotic (26), antioxidative (27) and anti-inflammatory effects (28). In addition, Lim et al., elucidated the anticancer and anti-atherosclerotic effects of trans-vaccenic acid, the main trans-C18:1 isomer in milk fat (29). CLA has been shown to be positively associated with bone mass in mice (30) and postmenopausal women (31).

### Milk Proteins

It is well-known that milk has more beneficial effects on bone health when compared to other food products. Milk is made up phosphoproteins such as growth factors, immunoglobulins, bovine serum albumin (BSA), alpha-lactalbumin and beta-lactoglobulin and a large quantity of caseins (32). Milk proteins are made up of bioactive peptides which are inactive or latent until released and activated by the process of enzymatic proteolysis during food processing, gastrointestinal digestion, fermentation of milk with proteolytic starter cultures or hydrolysis by proteolytic enzymes (33). Enzymatic proteolysis, for example, the actions of proteinases and peptidases from lactic acid bacteria are important for the release and activation of the milk-protein derived bioactive peptides known to be inactive within the sequence of the parent protein (34).

Casein is the major source of amino acids in the milk-peptide sequence. The active sequence size which may vary from 2 to 20 amino acid residues and many peptides are also known for its multi-functional properties (33). Digestive enzymes in the human gastrointestinal tract (GIT) contribute to the further breakdown of long casein-derived oligopeptides which may lead to the release of bioactive peptides. Casein phosphopeptides (CPP) prepared from cow's milk beta-casein is known to enhance the absorption of calcium by increasing the soluble calcium concentration in the small intestinal lumen (35). On liberation, these bioactive peptides may produce hormone-like activity acting as regulatory compounds (34). Milk whey protein, in particular the milk basic protein (MBP) fractions have also been shown to promote bone formation and increase bone mineral density in both *in vivo* and *in vitro* studies (36, 37). Caseinoglycomacropeptide (CGMP) is an acidic protein from milk released from kappa-casein during coagulation in the making of cheese. The milk acidic protein has also been reported to reduce bone loss in ovariectomised rats (38, 39). Lactic acid bacteria produce lactic acid whey by the process of fermentation in the making of cheese. However, the lactic acid whey and mineral acid whey do not contain CGMP (32).

Lactoferrin is known as an iron-binding glycoprotein found in milk which is capable of stimulating bone formation cells, osteoblasts while inhibiting the osteoclasts, bone resorption cells (40). Studies conducted *in vitro* (41) and *in vivo* (42) have shown a positive association and effects on bone mass in ovariectomized rats and mice. Osteopontin is another highly glycosylated and phosphorylated protein typically found in milk. It is a non-collagenous protein in bone which protects the exposed bone surface. Osteopontin is known to be important for bone cells and cell-matrix interactions (32).

## Pro-, Pre-, and Synbiotics

Milk products such as yogurt and kefir contain probiotics, however milk and dairy products may also be considered a good carrier of probiotics and prebiotics which are important for calcium absorption. Calcium bioavailability depends on various factors such as the source of calcium, age, transit time, the amount of calcium ingested, intestinal content, and type of diet. All of these factors play a role in the rate of calcium absorption. Calcium is a divalent cation which occurs as salt in food. Calcium gets absorbed in soluble form and must be in an ionized form (43). Prebiotics for example, improve mineral balance and bone properties by lowering cecal pH which can increase calcium solubility and absorption, increase in cecal wall thickness and content weight as well as increases in bifidobacterial (43).

### Benefit of Probiotics

Probiotics are known to contain living microorganism that tends to exert beneficial effects onto the GIT microbial population. The most commonly defined probiotic microbes are member of the genera *Lactobacillus, Bifidobacterium* as well as lactic acid and non-lactic acid bacteria (44). Probiotics has numerous advantages and functions in the human organism with the main advantage being the ability to maintain appropriate balance between pathogens and bacteria necessary for normal function. This positive effect is also used to restore the natural microbiota in the gut after antibiotic therapy (45).

Another role of the probiotics is in the counteraction of the activities of pathogenic intestinal bacteria which were caused by contaminated food and environment. Probiotic microbes have been reported to increase the immune system efficiency, enhance vitamin, and mineral absorption as well as generate organic acids and amino acids (46). Some probiotics may produce enzymes such as lipase and esterase and co-enzymes A, Q, NAD and NADP. All these have been shown to exhibit antibiotic (acidophiline, bacitracin and lactacin, a bacteriocin) (47), anti-cancerogenic (48), and immunosuppressive properties (49).

Bone health depends on the bone remodeling cycle and the balance of the bone formation by osteoblasts and bone resorption process by osteoclasts. These processes is regulated by hormones, immune cells, and the gastrointestinal system while the healthy intestine is known for mineral (calcium, phosphorus) absorption and subsequent bone mineralization (50). In addition, the intestine also produces endocrine factors such as incretins and serotonins that signal (crosstalk) to bone cells.

A randomized control trial have shown that *Lactobacillus reuteri* reduced bone loss in elderly women aged 75–80 years with low bone mineral density in Sweden (51). Similarly, a multispecies probiotic supplement (Gerilact capsule) slowed down the rate of bone turnover in postmenopausal women aged 50–72 years in Iran (52). Furthermore, when the probiotic is combined with bioavailable isoflavones, it improved estrogen metabolism and bone health status in postmenopausal women (53).

### Benefit of Prebiotics

Prebiotics are also known as functional food components that have a great potential of modulating the gut microbiota as well as the gut environment and pH. These non-digestible oligosaccharides containing prebiotic activities occur naturally in human milk and plants. Prebiotics either occur naturally in plant-based foods or by enzymatic conversion of sugars from synthetic production of carbohydrate structures or soluble dietary fibers (54).

Some prebiotics are artificially produced, they include lactulose, galactooligosaccharides (GOS), fructooligosaccharides (FOS), maltooligosaccharides (MOS), cyclodextrins, and lactosaccharose. Lactulose contains a significant part of oligosaccharides produced and can be as much as 40% content (55). Fructans, such as inulin and oligofructose, are believed to be the most used and effective in relation to many species of probiotics (46). Prebiotics in the colon are fermented by beneficial bacteria. This fermentation which may then lead to an increased production and/or a change in the relative abundance of SCFAs (acetate, butyrate and propionate) and lactate, moderate reduction in the colonic pH, increase in stool mass, reduction in nitrous end products and fecal enzymes, and a resulting improvement in the immune system (56). There are two mechanisms by which dietary fiber increases the fecal bulk: incompletely fermented types of fiber binding to water throughout the GIT, whereas the readily fermented types of fiber tend to increase the microbial mass (57).

Human studies have shown FOS (58), GOS (59) and xylooligosaccharide (XOS) (60) are compounds that are able to increase the proportion of *Bifidobacterium* in the GIT whereas the number of *Bacteroidaceae* and *Clostridium perfringens* are decreased. Another study with the intake of lactulose (2 × 10 g d^−1^) in a rat model showed increased probiotic bacteria and decreased putrefactive and potential pathogenic bacteria (61).

### Benefit of Synbiotics

Bacteria are known to produce genes in response to changes in their environment. These genes are responsible for encoding enzymes that produce metabolites such as short chain fatty acids (i.e., butyrate, lactate, propionate and acetate), branch-chained fatty acids, bile acid derivates and vitamins. These metabolic products are however dependent on the availability of substrate produced in part by prebiotics and the intestinal microbiota (62). Furthermore, prebiotics causes the lowering of intestinal pH and is also important for maintaining the osmotic retention of water in the bowel (46).

These synbiotics are also known to stimulate and result in enlarged absorption surface area by the proliferation enterocytes mediated by bacterial fermentation (63). Part of the activities of these microbes includes absorption and release of essential nutrients from the diet (mainly calcium and phosphate), regulation of the immune system, support of intestinal integrity and function as well as the ability to repress pathogenic invasion of harmful bacteria or colony (62). It also responsible for promoting the expression of calcium-binding proteins and degradation of mineral complexing molecules such as oxalates and phytic acid. The commensal bacteria have recently been also thought to affect the bone physiology through production of small molecules such as serotonin or estrogen-like molecules beneficial for osteogenesis.

A study conducted in healthy postmenopausal women showed that synbiotic fermented milk was capable of enhancing the oral bioavailability and plasma concentrations of isoflavones (64). Isoflavones are known to be able to alleviate the risk of osteoporosis.

## Discussion

In this review of the literature, we have evaluated the benefits of milk components, probiotics, prebiotics and synbiotics in calcium absorption and bone health. Dairy products especially milk is known to provide phosphorus intake simultaneously with calcium intake unlike supplementation. Likewise, milk/dairy do not contain calcium absorption inhibiting substances such as oxalates, phytates or polyphenols (65). Milk contains substantially high levels of vitamin D, riboflavin, vitamin C and vitamin B12 necessary for bone health maintenance. The consumption of milk is also known to influence hormones such as IGF-1 and the bone cytokines.

According to Goulding (66), CPPs increase calcium absorption by reducing the production of insoluble calcium phosphate complexes through signaling and biomodulatory effects in the GIT. In a systematic review of literature by Kouvelioti et al. (67), they concluded that the systematic consumption of dairy products may be beneficial for bone structure and development. Furthermore, a review by Heuvel and Steijns (68) revealed a strong evidence for the positive impact of calcium from dairy products with or without vitamin D on bone mineralization. Studies have also shown that calcium absorption may be mediated by the gut microbiota specifically by *Bifidobacteria* and *Lactobacillus*, and probably fermented milk products (69).

### Microbial Differences Between Healthy and Osteopenic/Osteoporotic Women

To our best knowledge, two human studies have investigated the role of gut microbiota in bone health. The two studies used 16S ribosomal RNA (rRNA) gene sequencing to characterize the microbiota composition profile. Wang et al. (70) found the relative abundance of *Blautia, Parabacteroides*, and *Ruminococcaceae* to be higher among the osteoporotic patients compared to their normal controls. This study was conducted with a small sample size of mainly female patients recruited from a Chinese hospital with mean age between 64 and 70 years. Das et al. (71) found that *Lactobacillus* and *Bacillus* was proportionally more abundant in participants with osteoporosis compared to those with normal BMD. This study involved 181 female and males aged between 55 and 75 years care referrals.

Our recent novel findings using shotgun metagenomics have shown alpha diversity differences between healthy (H) and osteopenic/osteoporotic (OP) postmenopausal women in New Zealand. The women were classified according to the World Health Organization (WHO) *T*-score cut-offs for diagnosing osteoporosis. The findings also showed a higher proportion of *Bacteroidetes Bacteroides* among the OP group compared to the H group. The result of the finding also indicated higher microbial diversity among the H group than the OP group. Lower microbial composition diversity in the women with bone disorders is in agreement with other studies where lower diversity has been observed in “disease” states vs. healthy controls. Limited published epidemiological evidence supports the relationship between the gut microbiota and bone mineral density and robust human studies are required to evaluate this relationship. Our results provide a basis for a longitudinal intervention assessment of the role of intestinal microbiota composition and function in osteoporosis to yield preventive and therapeutic interventions. In addition, further investigations using probiotic, prebiotic, or synbiotic foods in intervention studies may be able to provide more insight into the role of these foods in bone health maintenance.

## Conclusion

Milk and milk components have been shown to significantly affect growth and bone mineralization in children. Studies in older women using regular or fortified milk have reported significant changes in bone biomarkers and some changes in bone mineral density, though not reduction in fracture risk. Milk and dairy products also contain several other bioactives that can affect bone formation or resorption. Combining the inherent bioactives in milk with additional pre- or probiotics can enhance the beneficial effects of milk on bone health via improvement of the intestinal absorption of minerals especially calcium and magnesium. The observed differences in diversity of microbiota between healthy and osteoporotic women open new avenues for modulating the gut profile and thereby preventing bone loss or maintaining healthy bone density.

## Author Contributions

MK was responsible for the conceptualization, writing the abstract, introduction and conclusion while BI-O wrote the body of the article. Both authors read, reviewed, and approved the final draft.

## Conflict of Interest

The authors declare that the research was conducted in the absence of any commercial or financial relationships that could be construed as a potential conflict of interest.
